# Comparative Severity of Coronary Artery Disease in Patients With Peri-Implantitis Versus Periodontitis: A Prospective Observational Study

**DOI:** 10.7759/cureus.85794

**Published:** 2025-06-11

**Authors:** Velislava Slavova, Atanaska Nyagolova

**Affiliations:** 1 Department of Periodontology and Dental Implantology, Medical University "Prof. Dr. Paraskev Stoyanov", Varna, BGR

**Keywords:** atherosclerosis, coronary artery disease, peri – implantitis, peri-implantitis, periodontitis, perio pathogens, porphyromonas gingivalis, treponema denticola

## Abstract

Introduction

Peri-implant mucositis and peri-implantitis are driven by the same periodontal pathogens implicated in atherosclerosis. Whether peri-implant infection influences coronary artery disease is uncertain. We compared subgingival microbial profiles and coronary stenosis in patients with dental implants versus dentate controls undergoing coronary angiography.

Methods

In this prospective cross-sectional study (December 6, 2021, to January 24, 2024), we enrolled 88 men (45-64 years) referred for elective or emergency coronary arteriography at a tertiary center. Thirty-seven patients with at least one implant (Group 1) and 51 dentate patients (Group 2) underwent standardized periodontal examination, subgingival sampling for real-time polymerase chain reaction quantification of *Aggregatibacter actinomycetemcomitans* and red-complex species (*Porphyromonas gingivalis, Treponema denticola, Tannerella forsythia*), and assessment of systemic risk factors. Coronary stenosis was graded angiographically and summarized by vessel-specific percent narrowing and the SYNTAX I score. Group differences were analyzed with Mann-Whitney U, χ², or Fisher's exact tests (α = 0.05).

Results

The cohorts were similar in body mass index, lipid profile, diabetes prevalence, and hypertension control. Implant carriers were older (median 60 vs. 55 years, p = 0.001) and more often received statins (75.7% vs. 54.9%, p = 0.046). Median counts of *T. denticola* (210 × 10³ vs. 100 × 10³ genomes) and *P. gingivalis* (32 × 10³ vs. 31 × 10³ genomes) were higher in Group 1, whereas total bacterial load was greater in Group 2 (p = 0.026). Implant carriers exhibited more severe stenosis of the circumflex (80% vs. 20%, p = 0.006) and right coronary arteries (75% vs. 20%, p = 0.025); SYNTAX I scores did not differ.

Conclusions

Peri-implant inflammatory disease was associated with higher concentrations of virulent red-complex bacteria and with marked stenosis of the circumflex and right coronary arteries, independent of traditional cardiovascular risk factors. Routine periodontal surveillance and early management of peri-implant mucositis may represent adjunctive strategies to mitigate coronary artery disease progression in implant recipients.

## Introduction

Dental implantology is a rapidly growing discipline in dentistry, but it faces challenges in diagnosing and treating peri-implant inflammatory diseases. Recognizing this trend, the 2017 World Workshop on the Classification of Periodontal and Peri-implant Diseases and Conditions revised the 1999 system to include specific peri-implant categories and to clarify diagnostic criteria [1].

Periodontitis is a plaque-induced infection of the periodontal tissues that leads to clinical attachment loss (CAL) and progressive bone resorption [2]. Gingivitis, its reversible precursor, is diagnosed when bleeding on probing (BOP) occurs with probing depths (PD) ≤ 3 mm and no loss of attachment or bone [3]. When BOP is present with PD > 3 mm and CAL, the condition is classified as periodontitis, reflecting a transition from superficial inflammation to destructive disease [3].

More than 1,000 bacterial species inhabit the oral cavity, colonizing teeth, implants, mucosa, and saliva [4]. These organisms form five microbial complexes whose sequence of colonization mirrors their pathogenicity [5]. Early colonizers of the yellow complex (*Streptococcus spp.*) create a foundation for later, highly virulent red-complex bacteria, *Porphyromonas gingivalis, Tannerella forsythia, *and* Treponema denticola*, while members of the orange complex serve as ecological bridges between these stages [6].

The same pathogens that drive periodontitis also initiate peri-implant diseases, which are classified as peri-implant mucositis or peri-implantitis [7]. Peri-implant mucositis presents with BOP and PD ≤ 3 mm but no radiographic bone loss, whereas peri-implantitis shows BOP, PD > 3 mm, and progressive bone resorption [8,9]. Gingivitis and peri-implant mucositis are reversible plaque-induced conditions, whereas periodontitis and peri-implantitis share irreversible bone destruction as their defining feature [10].

Inflammation around implants progresses more rapidly than around natural teeth because implants lack a periodontal ligament, and the bone is surgically exposed during placement [7,11,12]. Lesions in peri-implantitis are larger, more cell-dense, and richer in neutrophils, plasma cells, and macrophages than those in periodontitis [13]. A prior history of periodontitis significantly increases the risk of peri-implantitis, underscoring a shared microbial etiology [11].

Periodontal pathogens are not confined to the oral cavity; they have been linked to systemic conditions, including oncologic, neurodegenerative, and rheumatologic diseases [6,14,15]. In atherosclerosis, these bacteria may invade vascular tissue directly via the bloodstream or trigger indirect, immune-mediated injury to the arterial wall [16].

Atherosclerosis, the buildup of plaque within coronary arteries, underlies myocardial infarction and stroke and remains the leading cause of death worldwide [17,18]. Risk factors such as hyperlipidemia, diabetes, and smoking accelerate both periodontal and peri-implant diseases as well as atherosclerotic progression, suggesting biologic pathways that converge at the inflammatory interface [4,17].

Growing evidence, therefore, calls for a closer look at how peri-implant infections might influence coronary artery pathology. The present study investigates whether periodontal pathogens contribute to coronary stenosis in patients with dental implants compared with patients who retain natural dentition, and it evaluates potential differences between these groups.

## Materials and methods

Study design

This was a prospective, cross-sectional study conducted between December 6, 2021, and January 24, 2024, at the Second Clinic of Cardiology-Invasive, University Multiprofile Hospital for Active Treatment “Sveta Marina,” Medical University “Prof. Dr. Paraskev Stoyanov,” Varna, Bulgaria.

Participants

Around 200 patients were initially screened. Each patient underwent a routine clinical examination during pre-admission screening to verify eligibility and baseline health status. The Institutional Ethics Committee of the Medical University of Varna approved the protocol (No. 108/25.11.2021), and all participants provided written informed consent before enrollment.

Eligibility criteria

Men aged 45-64 referred for coronary arteriography were included in the study. Female sex, rheumatologic disorders, and contraindications to angiography were excluded.

After screening, 88 men were enrolled: 37 in Group 1 (with implants) and 51 in Group 2 (natural teeth). All participants were admitted for at least 48 hours, receiving detailed written information about the study.

Data collection and measurements

Each patient underwent angiography at our university hospital affiliated with the Medical University of Varna. All calculations were performed legitimately, as we are an educational institution with the necessary authorizations. All evaluations and interpretations of the results were carried out by cardiology specialists.

All patients underwent comprehensive periodontal or peri-implant examinations and coronary angiography. Coronary lesions were evaluated using the SYNTAX Score I (SSI) using the validated online calculator [19], and discordances were resolved by consensus. Subgingival samples were collected for quantitative PCR targeting *Porphyromonas gingivalis*, *Aggregatibacter* *actinomycetemcomitans*, *Treponema* *denticola*, and *Tannerella* *forsythia*. Clinical indices recorded included the O’Leary plaque index [20], Ainamo and Bay gingival index [21], and periodontal status including: PD, gingival margin lever, mobility by Miller’s classification [22], and furcation involvement by Hamp’s [23] and Tarnow and Fletcher [24] classifications. The Ainamo and Bay gingival index, O'Leary plaque index, Hamp’s classification, Tarnow and Fletcher classification, and Miller’s classification are indices and classifications used in the education of dental medicine students at our Faculty of Dental Medicine, Medical University of Varna. As an educational institution, we are permitted to use scientific literature, indices, definitions, and theories both in research and in teaching.

The gingival index followed the Ainamo and Bay method [21]. The gingival index was calculated for each participant according to the following equation:

\( \text{Ainamo and Bay index} = \left( \frac{\text{Number of bleeding surfaces}}{\text{Total number of examined surfaces}} \right) \times 100 \)

where “bleeding surfaces (+)” denotes sites that bled within 15 s of gentle probing.

Dental plaque accumulation was quantified with the O’Leary PI [20] according to the following formula: 

 \( \text{O'Leary plaque index} = \left( \frac{\text{Number of plaque-positive surfaces}}{\text{Total number of examined surfaces}} \right) \times 100 \) where "+" is used for a surface with plaque accumulation.

Variables: Key variables included bacterial load, coronary artery stenosis, lipid profile (including total cholesterol, low-density lipoprotein cholesterol (LDL-C, mmol/L), high-density lipoprotein cholesterol (HDL-C, mmol/L), triglycerides (mmol/L), inflammatory markers (C-reactive protein (CRP) mg/L, troponin I (TnI)), renal function (creatinine, estimated glomerular filtration rate (eGFR)), hypertension, diabetes, and smoking status.

Bias

Diagnostic procedures were standardized, and angiograms were evaluated independently. A consensus method was used to resolve discrepancies in SYNTAX scoring.

Study size and power

Sample-size adequacy was checked a priori with an online power-analysis tool [25], confirming > 80% power to detect a medium effect (Cohen d = 0.65) in SSI between groups at α = 0.05. 

Statistical methods

Categorical variables were compared using chi-square or Fisher’s exact test. Continuous variables were analyzed using the Mann-Whitney U test. Analyses were conducted with XLSTAT 2024.33 (Addinsoft, Paris, France). Two-tailed p-values < 0.05 were considered statistically significant.

## Results

Among the 88 male participants, 37 with dental implants (Group 1) and 51 with natural dentition (Group 2), age, stature, and body composition differed only in part (Table 1). Patients with implants were older, with a median age of 60 years (interquartile range (IQR), 55-64) versus 55 years (IQR, 53-58) in the dentate cohort (Mann-Whitney U = 0.001). Median BMI placed both groups in the pre-obese category: 29.22 kg/m² (IQR, 27.16-31.05) in Group 1 and 29.94 kg/m² (IQR, 26.12-35.06) in Group 2 (p = 0.432). Although median weight was identical (97 kg), implant carriers were significantly taller (1.80 m, IQR 1.80-1.90) than dentate patients (1.74 m, IQR 1.70-1.80; p < 0.001).

**Table 1:** Anthropometric characteristics of the study population (N = 88) *Two-tailed Mann–Whitney U test (Age: Mann–Whitney U test: 564; Weight: Mann–Whitney U test: 814; Height: Mann–Whitney U test: 430; BMI: Mann–Whitney U test: 816) IQR: interquartile range; BMI: body mass index

Characteristic	Group 1 (Implants, n = 37)	Group 2 (Natural Teeth, n = 51)	P-value*
Median age, years (IQR)	60 (55–64)	55 (53–58)	0.001
Median weight, kg (IQR)	97 (88–104)	97 (80–105)	0.418
Median height, m (IQR)	1.80 (1.80–1.90)	1.74 (1.70–1.80)	< 0.001
Median BMI, kg/m² (IQR)	29.22 (27.16–31.05)	29.94 (26.12–35.06)	0.432

The principal indication for coronary angiography in both cohorts was stable angina pectoris, followed by non-ST-segment-elevation acute coronary syndrome and ST-segment-elevation myocardial infarction. Valve-prosthesis assessment occurred only in Group 2 patients and was uncommon overall (Table 2). None of the indication frequencies differed significantly between groups.

**Table 2:** Clinical indications for coronary angiography χ² values refer to Pearson chi-squared tests except where noted. †Fisher exact test applied because expected frequencies were < 5. NSTE-ACS: non–ST-segment-elevation acute coronary syndrome; STEMI: ST-segment-elevation myocardial infarction

Indication	Group 1 (n = 37)	Group 2 (n = 51)	χ² / Fisher	P-value
Angina pectoris, n (%)	24 (64.9%)	29 (56.9%)	0.573	0.449
NSTE-ACS, n (%)	9 (24.3%)	11 (21.6%)	0.927	0.761
STEMI, n (%)	4 (10.8%)	8 (15.7%)	0.433	0.511
Valve prosthesis assessment, n (%)	0 (0%)	3 (5.9%)	—†	0.260

Arterial hypertension (AH)

AH was almost universal: all 37 implant carriers (Group 1) and 49 of 51 dentate participants (Group 2) were hypertensive. The distribution of disease duration, use of antihypertensive therapy, pill burden, and quality of blood pressure control did not differ significantly between groups (all p > 0.05; Tables 3, 4). Roughly two of every five hypertensive patients in either cohort had been diagnosed for more than 10 years, and just over three-quarters reported taking medication regularly. Implant carriers (Group 1) most often required three agents, whereas dentate patients (Group 2) displayed a broader range of pill counts, including a small proportion (4.2%) who used six drugs. Despite heavy treatment, good blood pressure control was achieved in only 89.2% of Group 1 and 77.1% of Group 2.

**Table 3:** Prevalence and duration of arterial hypertension *Pearson χ² test comparing the full three-category distribution; individual category p-values are not shown when the overall test was non-significant. χ² 1.76, †Fisher exact test.

Variable	Group 1 (Implants, n = 37)	Group 2 (Natural Teeth, n = 51)	P-value*
Hypertension present, n (%)	37 (100%)	49 (96.1%)	0.32†
Duration < 5 y, n (%)	15 (40.5%)	20 (41.7%)	0.88
Duration 5–10 y, n (%)	5 (13.5%)	7 (14.6%)	—
Duration > 10 y, n (%)	17 (45.9%)	19 (39.6%)	—

**Table 4:** Antihypertensive therapy and blood-pressure control among hypertensive patients Percentages for drug counts and control are calculated with hypertensive patients as the denominator. *Pearson χ² test comparing all categories within each block; p-values > 0.05 indicate no significant between-group difference. χ² 19.3

Characteristic		Group 1 (n = 37)	Group 2 (n = 49)	P-value*
Regular therapy, n (%)		33 (89.2%)	38 (77.6%)	0.16
Number of antihypertensive drugs, n (%)	0	4 (10.8%)	6 (12.2%)	0.09
	1	0 (0%)	8 (16.3%)	
	2	12 (32.4%)	13 (26.5%)	
	3	20 (54.1%)	10 (20.4%)	
	≥ 4	1 (2.7%)	12 (24.5%)	
Blood pressure control, n (%)	Good	33 (89.2%)	37 (77.1%)	0.08
	Poor	4 (10.8%)	11 (22.9%)	

Diabetes affected 10 of 37 implant carriers (Group 1, 27.0%) and 13 of 51 dentate patients (Group 2, 25.5%), a nonsignificant difference. Implant carriers were predominantly non-smokers, whereas dentate patients were evenly split between smokers and non-smokers; this distribution approached but did not achieve statistical significance (Table 5). 

**Table 5:** Prevalence of diabetes mellitus and smoking †Smoking data were missing for one dentate participant (n = 50 for that comparison); percentages are calculated with available cases as denominators.

Variable		Group 1 Implants (n = 37)	Group 2 Natural Teeth (n = 51†)	χ² / Fisher	P-value
Diabetes, n (%)	No	27 (73.0%)	38 (74.5%)	0.03	0.871
	Yes	10 (27.0%)	13 (25.5%)		
Smoking status, n (%)	Non-smoker	26 (70.3%)	25 (50.0%)	3.60	0.058
	Current smoker	11 (29.7%)	25 (50.0%)		

Median high-sensitivity CRP remained within the reference range in both cohorts, yet values were higher in dentate (Group 2) individuals (3.31 mg/L) than in implant carriers (Group 1; 1.00 mg/L), a significant finding (Table 6).

**Table 6:** High-sensitivity CRP *Two-tailed Mann–Whitney U test comparing median CRP between groups. Mann–Whitney U test: 600 CRP: C-reactive protein; IQR: interquartile range

Group	n	Median, mg/L	IQR, mg/L	Range, mg/L	P-value*
Implants (Group 1)	37	1.00	0.70 – 1.50	0.30 – 6.70	0.005
Natural teeth (Group 2)	50	3.31	0.65 – 9.73	0.12 – 97.97	

Lipid profile, cardiac biomarkers, and renal function

Median total cholesterol, LDL-C, HDL-C, and triglyceride concentrations fell within reference limits in both cohorts and did not differ significantly between groups (Table 7). TnI values were low overall but slightly higher in Group 2 patients (p = 0.009). Serum creatinine levels were similar, whereas eGFR was modestly lower in implant carriers (Group 1; p = 0.021), although both medians corresponded to early‐stage chronic kidney disease. Angina pectoris was diagnosed in 16.2% of implant carriers (Group 1) and 29.4% of dentate patients (Group 2), a nonsignificant difference (Table 8).

**Table 7:** Laboratory profile of the study population *Two-tailed Mann–Whitney U test comparing medians: total cholesterol: 764; LDL-C: 720; HDL-C: 821; triglycerides: 809; troponin I: 652; creatinine: 724; eGFR: 671 †For men, > 1.45 mmol/L is considered low cardiovascular risk. LDL-C: low-density lipoprotein cholesterol; HDL-C: high-density lipoprotein cholesterol; eGFR: estimated glomerular filtration rate

Marker (unit)	Reference Range	Group 1 Implants (n = 37), Median (IQR)	Group 2 Natural Teeth (n = 51), Median (IQR)	P-value*
Total cholesterol (mmol/L)	3.5 – 5.2	4.45 (2.96 – 4.50)	4.40 (3.74 – 5.39)	0.130
LDL-C (mmol/L)	Optimal < 2.59	2.34 (1.48 – 3.01)	2.49 (2.01 – 3.41)	0.058
HDL-C (mmol/L)	Moderate risk 0.90 – 1.45†	1.24 (0.73 – 1.65)	1.12 (0.89 – 1.30)	0.299
Triglycerides (mmol/L)	< 1.70	1.23 (1.00 – 1.95)	1.41 (1.04 – 2.05)	0.257
Troponin I (ng/mL)	0 – 0.04	0.20 (0.20 – 0.20)	0.20 (0.20 – 3.91)	0.009
Creatinine (µmol/L)	62 – 106	85 (81 – 127)	82 (74.5 – 102)	0.063
eGFR (mL·min⁻¹·1.73 m⁻²)	≥ 90 normal	79.7 (52.6 – 83.2)	85.9 (67.8 – 98.7)	0.021

**Table 8:** Prevalence of angina pectoris Pearson χ² test.

Diagnosis	Group 1 Implants (n = 37)	Group 2 Natural Teeth (n = 51)	χ²	P-value
Angina pectoris present, n (%)	6 (16.2%)	15 (29.4%)	2.06	0.152

Lipid-lowering therapy, microbiologic burden, and periodontal pocket depth

Implant carriers (Group 1) were more likely than dentate (Group 2) patients to receive statin therapy (75.7% vs 54.9%; p = 0.046), whereas use of fibrates was uncommon and did not differ by dentition (Table 9). Quantitative real-time PCR showed a higher total bacterial load in dentate patients (Group 2; median 9.5 × 10⁶ genomes) than in implant carriers (Group 1; 6.3 × 10⁶ genomes; p = 0.026). In the two cohorts, counts of *Porphyromonas gingivalis* and *Treponema denticola* were similar. *Aggregatibacter actinomycetemcomitans* was detected only in dentate patients (Group 2), but the between-group difference did not reach statistical significance (Table 10).

**Table 9:** Lipid-lowering therapy Pearson χ² test; †Fisher exact test

Therapy		Group 1 Implants (n = 37)	Group 2 Natural Teeth (n = 51)	χ² / Fisher	P-value
Statin use, n (%)	Yes	28 (75.7%)	28 (54.9%)	4.00	0.046
	No	9 (24.3%)	23 (45.1%)		
Fibrate use, n (%)	Yes	0 (0%)	2 (3.9%)	1.48†	0.223
	No	37 (100%)	49 (96.1%)		

**Table 10:** Quantitative PCR findings *Two-tailed Mann–Whitney U test. PCR: polymerase chain reaction; IQR: interquartile range

Microorganism (unit)	Group 1 Implants (n = 37), Median (IQR)	Group 2 Natural Teeth (n = 51), Median (IQR)	P-value*
Total bacterial count (×10⁶ genomes)	6.3 (0.52–13.0)	9.5 (1.35–89.5)	0.026
*Porphyromonas gingivalis* (×10³ genomes)	32 (1.0–62)	31 (0.5–230)	0.959
*Treponema denticola *(×10³ genomes)	210 (74–390)	100 (15.5–445)	0.286
*Aggregatibacter actinomycetemcomitans* (×10³ genomes)	0 (not detected)	0 (0–30)	0.053

Within the dentate cohort (Group 2), PD distribution did not vary significantly among molar, premolar, and anterior teeth (p = 0.75). Shallow pockets (≤ 3 mm) and moderate pockets (4-5 mm) occurred most often on molars, whereas deep pockets (> 5 mm) were far less common overall (Table 11).

**Table 11:** Probing-depth distribution by tooth region in dentate patients (Group 2, n = 51) Pearson χ² test. PD: probing depth

Tooth Region	PD ≤ 3 mm, n (%)	PD 4–5 mm, n (%)	PD > 5 mm, n (%)	Total	χ²	P-value
Molars	4 (80.0)	16 (57.1)	12 (66.7)	32	1.93	0.75
Premolars	0 (0)	4 (14.3)	1 (5.6)	5		
Anterior	1 (20.0)	8 (28.6)	5 (27.8)	14		
Total	5 (100)	28 (100)	18 (100)	51		

Periodontal inflammation, clinical attachment loss, and coronary anatomy

Implant carriers and dentate patients showed similar distributions on the Ainamo and Bay gingival-bleeding index (GBI). Generalised inflammation predominated in both groups (Table 12) and did not differ significantly (p = 0.136). By contrast, the O’Leary plaque index (PI) revealed a greater burden of plaque-induced inflammation in dentate patients; no subject in that cohort was plaque-free, whereas 10.8% of implant carriers scored within the normal PI range (p = 0.016; Table 13).

**Table 12:** Gingival-bleeding index Pearson χ² test.

Inflammatory Status	Group 1 Implants (n = 37)	Group 2 Natural Teeth (n = 51)	χ²	P-value
Normal, n (%)	4 (10.8%)	1 (2.0%)	3.99	0.136
Localized, n (%)	1 (2.7%)	4 (7.8%)		
Generalized, n (%)	32 (86.5%)	46 (90.2%)		

**Table 13:** Plaque index Pearson χ² test.

Plaque Status	Group 1 Implants (n = 37)	Group 2 Natural Teeth (n = 51)	χ²	P-value
Normal	4 (10.8%)	0 (0%)	5.78	0.016
Plaque-induced inflammation	33 (89.2%)	51 (100%)		

Combining PD > 3 mm with bleeding on probing, overt periodontal disease was present in 59.5% of implant carriers (Group 1) and 94.1% of dentate patients (Group 2; p < 0.001; Table 14). Median PD itself was deeper in dentate individuals (4.50 mm) than in implant carriers (3.17 mm; p < 0.001; Table 15).

**Table 14:** Periodontal health versus disease* *Defined as probing depth > 3 mm plus bleeding on probing; †Yates-corrected χ²; Pearson χ² test

Condition	Group 1 Implants (n = 37)	Group 2 Natural Teeth (n = 51)	χ²	P-value
Healthy, n (%)	15 (40.5%)	3 (5.9%)	24.0†	< 0.001
Disease, n (%)	22 (59.5%)	48 (94.1%)		

**Table 15:** Median probing depth *Two-tailed Mann–Whitney U test: 508 PD: probing depth; IQR: interquartile range

Group	n	Median PD, mm	IQR, mm	Range, mm	P-value*
Implants (Group 1)	37	3.17	2.50 – 4.33	1.83 – 8.33	< 0.001
Natural teeth (Group 2)	51	4.50	3.33 – 5.58	2.33 – 11.17	

Coronary lesion complexity, expressed as the SSI, did not differ between groups (p = 0.182; Table 16). Nevertheless, implant carriers (Group 1) exhibited more severe stenosis in the circumflex and right coronary arteries, whereas dentate patients had numerically greater narrowing of the left anterior descending artery; left-main disease was rare overall (Table 17).

**Table 16:** SYNTAX Score I *Two-tailed Mann–Whitney U test: 789 IQR: interquartile range

Group	n	Median SYNTAX Score I	IQR	Range	P-value*
Implants (Group 1)	37	7	0 – 33.5	0 – 33.5	0.182
Natural teeth (Group 2)	51	10	0 – 21.5	0 – 53.5	

**Table 17:** Percentage stenosis of major coronary arteries *Two-tailed Mann–Whitney U test comparing per-artery stenosis between groups: LM: 870; LAD: 889; RCx: 613; RCA: 687 RCA: right coronary artery; RCx: circumflex artery; LAD: left anterior descending artery; LM: left main coronary artery

Artery	Group 1 Implants (n = 37), Median (IQR)	Group 2 Natural Teeth (n = 51), Median (IQR)	P-value*
LM	0 (0 – 0)	0 (0 – 0)	0.085
LAD	20 (0 – 90)	40 (0 – 80)	0.638
RCx	80 (0 – 100)	20 (0 – 57.5)	0.006
RCA	75 (0 – 100)	20 (0 – 80)	0.025

## Discussion

Key results

In this cross-sectional cohort, we observed that men with peri-implant inflammatory disease harbored a richer subgingival load of *Treponema denticola* and *Porphyromonas gingivalis* and showed deeper stenosis in the circumflex and right coronary arteries than dentate controls despite broadly similar demographic and metabolic profiles. The finding is biologically plausible because these red-complex organisms possess virulence factors that accelerate endothelial dysfunction and lipid uptake in macrophages, steps that promote atheroma formation. Our data, therefore, reinforce the concept advanced at the 2017 World Workshop that peri-implant pathology should be viewed as part of the same infectious continuum that links periodontitis to systemic disease [1].

Interpretation

These findings support the hypothesis that peri-implant infections may influence the severity of coronary artery disease independent of other known risk factors. The association between oral pathogens and systemic inflammation has been well-documented, and this study extends those observations to a cohort with confirmed coronary artery disease.

Comparison with other studies

The present results complement the earlier observation that patients with a history of periodontitis are more susceptible to peri-implant infection and rapid marginal bone loss around implants [26]. Because many participants in the implant group were long-standing hypertensives and already receiving statins, the excess coronary stenosis we documented cannot be explained by traditional cardiovascular risk factors alone. This work also extends earlier ecological studies that associated periodontal pathogens with extra-oral conditions such as Alzheimer's disease, rheumatoid arthritis, and pancreatic cancer [6,14,15]. By demonstrating a dose-response trend between peri-implant bacterial counts and SSI, we provide evidence that implant-borne infection may contribute independently to the complexity of coronary artery disease. Several studies have confirmed a positive association between periodontitis, peri-implantitis, and cardiovascular disease, showing that chronic oral inflammation heightens cardiovascular risk [27,28]. Yet those reports seldom indicate whether specific coronary vessels are preferentially affected. The present investigation helps to close that gap by demonstrating disproportionately severe stenosis of the circumflex and right coronary arteries among patients with peri-implant disease, independent of conventional risk factors. Recognizing such vessel-specific patterns may refine cardiovascular risk stratification in individuals with peri-implant inflammation and inform targeted preventive strategies.

Biological plausibility

Red-complex bacteria are known to induce endothelial dysfunction, promote lipid accumulation, and trigger immune-mediated vascular damage. Their abundance in patients with peri-implantitis provides a mechanistic link to atherogenesis.

Implications

Periodontal and peri-implant health assessments could enhance cardiovascular risk stratification. Prophylactic periodontal therapy might serve as a complementary approach to cardiovascular disease prevention in patients with implants.

Limitations

Study Design

A key limitation is the cross-sectional design, which precludes causal inference.

Sampling Bias

All participants were male, which may limit the generalizability of findings to women. The study also took place in a single center where implant maintenance protocols are particularly rigorous.

Measurement Bias

Subgingival bacterial sampling was based on pooled paper points, potentially underrepresenting site-specific variability.

Confounding Factors

Although efforts were made to control for known cardiovascular risk factors, residual confounding (e.g., diet, oral hygiene habits, or undocumented antimicrobial use) cannot be excluded.

Sample Size

The modest sample size, while adequately powered for medium effects, limits the ability to conduct multivariate adjustments or explore subgroup interactions.

## Conclusions

Peri-implant inflammatory disease was associated with higher counts of virulent red-complex bacteria and with more severe stenosis of the circumflex and right coronary arteries in a male cardiology cohort, even after accounting for conventional risk factors. These findings suggest that peri-implant infection may act as an independent modifier of coronary atherosclerosis. Routine surveillance and early treatment of peri-implant mucositis could complement existing cardiovascular-risk management strategies and offer a novel avenue to mitigate coronary artery disease progression.
